# Ileal transposition helps to regulate plasma hepatokine levels in obese Zucker (Crl:ZUC(ORL)-Lepr^fa^) rats

**DOI:** 10.1038/s41598-021-87293-w

**Published:** 2021-04-08

**Authors:** Dominika Stygar, Tomasz Sawczyn, Agnieszka Dulska, Elżbieta Chełmecka, Łukasz Mielańczyk, Natalia Matysiak, Michał Kukla, Maciej Idzik, Jan Augustyniak, Andrzej Gabriel, Konrad Karcz, Jerzy Jochem

**Affiliations:** 1 Department of Physiology, Faculty of Medical Sciences in Zabrze, Medical University of Silesia, Katowice, Poland; 2 Department of Instrumental Analysis, Faculty of Pharmaceutical Sciences in Sosnowiec Medical University of Silesia, Katowice, Poland; 3 Department of Histology and Cell Pathology, Faculty of Medical Sciences in Zabrze, Medical University of Silesia, Katowice, Poland; 4 Department of Endoscopy, The University Hospital in Cracow, Cracow, Poland; 5 Independent Public Health Care, Opole Cancer Center Prof. Tadeusz Koszarowski, Opole, Poland; 6 Clinic of General, Visceral, Transplantation and Vascular Surgery, Hospital of the Ludwig Maximilian University, Munich, Germany

**Keywords:** Physiology, Metabolism, Metabolic diseases, Obesity

## Abstract

We studied the long-term effect of ileal transposition (IT) metabolic surgery on the hepatokines: retinol-binding protein-4 (RBP4), α-2-HS-glycoprotein (aHSG/fetuin-A), and fibroblast growth factor 21 (FGF21), C-reactive protein (CRP) plasma levels, glucose metabolism, body weight, liver histology, as well as total lipids concentration in muscle, liver, and fat tissue of obese Zucker (Crl:ZUC(ORL)-Lepr^fa^) rats. 14 adult males were randomly submitted either to IT or SHAM (control) surgery. Pre-operative hepatokines plasma levels were not significantly different in rats submitted to IT or SHAM protocol. Three months after the procedures the plasma levels of RBP4, aHSG, FGF21, and CRP were significantly lower in IT-operated animals when compared to SHAM-operated group. Three and 12 weeks after the IT and SHAM surgery, the AUC_OGTT_ were significantly lower than AUC_OGTT_ before the surgery. HOMA-IR was lower in rats after IT surgery in comparison to the SHAM-operated rats. Muscle and liver total lipids concentration was reduced after the IT procedure when compared to pre-IT conditions. IT had a significant reductive impact on the body weight in comparison to SHAM surgery in the 4th, 6th, 8th, and 10th week after the surgery. We conclude that IT reduces hepatokines’ plasma concentrations, muscle and liver total lipids concentration but not the inflammatory processes in the liver of Zucker (Crl:ZUC(ORL)-Lepr^fa^) rats.

## Introduction

Bariatric surgery is the most clinically effective treatment of severe and complex obesity both in terms of weight loss and improvement of weight-related diseases^1–3^. It induces many changes in patients’ lives, including changes in daily physical activity, physical and mental health, eating behaviour and food relations, social relations, sex life and body image^4^.

Type 2 diabetes mellitus (T2DM) has a very complex pathogenesis. In this study, we focused on T2DM and its associations with selected parameters: retinol-binding protein-4 (RBP4), α-2-HS-glycoprotein (aHSG/fetuin-A), fibroblast growth factor 21 (FGF21), and C-reactive protein (CRP). Plasma concentrations of RBP4, which is considered as an adipocyte-derived marker of T2DM^5,6^, are two to three-fold higher in overweight and obese subjects in comparison to the control group with normal body weight^7^. RBP4 plasma levels positively correlate with adipose RBP4 mRNA concentration, intraabdominal fat mass, body mass index (BMI) and insulin resistance^7^. aHSG/fetuin-A is an insulin receptor inhibitor that is produced in the liver and an important link between obesity and insulin resistance^8,9^. It is considered as an independent risk factor of T2DM which concentration increases even in prediabetic subjects^10^. aHSG levels correlate with fatty liver mass and atherogenic lipid profiles both in diabetic and nondiabetic models, regardless of the weight^11^. FGF21 is a known regulator of glucose and lipid metabolism. It is produced in the liver, pancreas, skeletal muscles and adipose tissue^12–15^. FGF21 ameliorates hepatic gluconeogenesis and insulin resistance. It also enhances the lipolysis, while suppressing the liver lipogenesis, and promotes fatty acid oxidation^13,16^ that is particularly intensified in patients with morbid obesity and T2DM^12^. Morbid obesity leads to generalized low-grade chronic inflammation, which is reflected by an increased level of C-reactive protein (CRP)^17,18^. CRP is also considered an independent marker of non-alcoholic fatty liver disease (NAFLD); the monitoring of its plasma level allows assessing the reduction in severe inflammatory activity and steatosis in NAFLD patients after a significant weight loss^19^. The evident decrease in inflammatory parameters occurred after significant weight loss and was related to the postoperative period, i.e. > 6 months after laparoscopic sleeve gastrectomy^20,21^. NAFLD is characterized by an excessive fat accumulation in the hepatocytes of people who do not consume alcohol and presents as a wide range of asymptomatic diseases, steatosis necroinflammatory forms of the disease or non-alcoholic steatohepatitis (NASH). The latter one is characterized by varying degrees of steatosis, cytoskeletal damage (hepatocellular ballooning), and lobular inflammation with or without fibrosis^22^.

The effectiveness of bariatric protocol differs between types of bariatric surgery^23^. Therefore, the continuous research on the metabolic shifts and their pathophysiological mechanisms after different types of bariatric surgery is still needed. Despite wide research in the animal model, the effects of ileal transposition, type of bariatric surgery, are not fully studied yet but based on the current knowledge^24–26^, we hypothesised that IT would have a beneficial effect on the glucose metabolism and inflammatory processes measured by selected hepatokines. We studied the effects of IT protocol on RBP4, aHSG/fetuin-A, FGF21, and CRP levels, liver histology, HOMA-IR as well as total lipid content in the obese Zucker (Crl:ZUC(ORL)-Lepr^fa^) rats—an animal model of insulin resistance, glucose intolerance, metabolic syndrome, and genetic obesity^27^.

## Results

### Post surgery body weight changes

**Figure 1:**
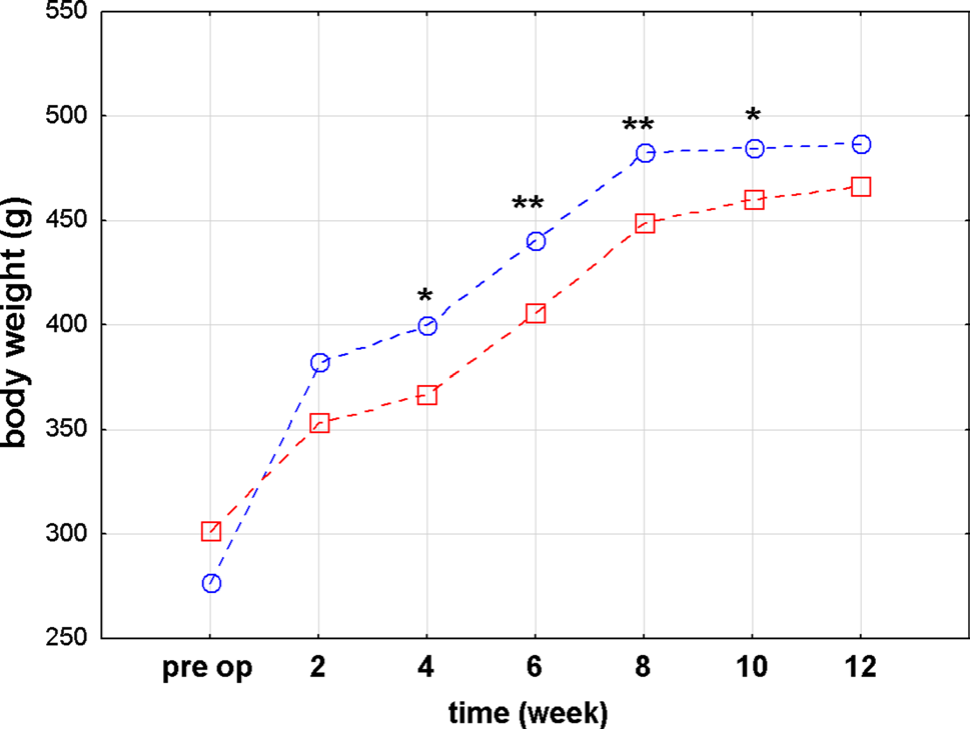
Comparison of body weight (g) of Zucker (Crl:ZUC(ORL)-Lepr^fa^) rats before surgery and every two weeks after ileal transposition (IT) or control (SHAM) surgery. For readers convenience, the points are connected with dashed lines and additionally significance level for p < 0.05 was marked as * and p < 0.01 as **, respectively.

Regardless of the type surgery performed—IT or SHAM—statistically significant differences in the body weight of rats were found and these differences depended on the time after surgery elapsed. Rats that underwent IT surgery had significantly reduced body weight at 4th, 6th, 8th and 10th weeks post operation in comparison to rats after SHAM procedure (p < 0.05, p < 0.001, p < 0.001, p < 0.05, respectively; Fig. 1). None of the selected surgical protocols had an impact on body weight gain after the procedure (p = 0.400). Body weight increase after the IT and sham surgery what is also connected with the fact that all rats were submitted in the age 7 weeks and they were still growing up. We have investigated the correlations between plasma parameters and the body weight. Due to the small size of the study groups, we calculated Spearman's rank-order correlation. The results of the analysis showed no statistically significant correlations between studied parameters.

### Hepatokines levels before and after ileal transposition (IT) and control (SHAM) surgery

#### Inter-group comparison

Retinol-binding protein-4 (RBP4), α-2-HS-glycoprotein (aHSG/fetuin-A), and fibroblast growth factor 21 (FGF21) plasma concentrations were the same for rats before ileal transposition (pre IT) and control (pre SHAM) surgery (Table 1). C-reactive protein (CRP) plasma concentration was significantly higher in rats selected for IT surgery (pre IT). Since the conditions and diet for both study groups were the same, this result reflects individual differences in the CRP levels in the population of rats used in the experiment (Table 1).

**Table 1:** Comparison of hepatokines: retinol-binding protein-4 (RBP4), α-2-HS-glycoprotein (aHSG/fetuin-A), fibroblast growth factor 21 (FGF21), and C-reactive protein (CRP) serum levels in blood of Zucker (Crl:ZUC(ORL)-Lepr^fa^) rats before and after control (SHAM) surgery or ileal transposition (IT).

Hepatokine concentration (ng/ml)	SHAM	IT	p
**Pre-operation**			
RBP4	3.4 ± 0.6	3.6 ± 0.5	0.781
aHSG	23.9 ± 3.3	21.6 ± 2.3	0.203
FGF21	2.4 ± 0.7	1.9 ± 0.5	0.196
CRP	16.0 ± 2.8	24.4 ± 2.3	**< 0.001**
**Post-operation**			
RBP4	3.3 ± 0.6	2.5 ± 0.3	**< 0.05**
aHSG	25.7 ± 3.4	19.9 ± 1.3	**< 0.01**
FGF21	2.0 ± 0.5	1.3 ± 0.5	**< 0.05**
CRP	19.4 ± 3.0	16.1 ± 1.8	**< 0.001**

The results are presented as mean ± standard deviation.

Significance for p-value was set at < 0.05.

Three months after IT and SHAM (control) procedures, the concentrations of RBP4, aHSG (fetuin-A), and FGF21 measured in plasma were significantly lower in rats after IT surgery when compared to animals after the SHAM procedure (Table 1). Also, CRP plasma levels were significantly lower in rats after IT surgery than in rats after control (SHAM) procedure (Table 1).

#### Intra-group comparison

Three months after the procedures, RBP4 concentration in rats after IT was significantly lower than in SHAM-operated animals. There were no differences concerning other hepatokines concentrations between the groups at that timepoint (Table 2). This can result from individual differences between animals included in the study.

**Table 2:** Comparison of hepatokines: retinol-binding protein-4 (RBP4), α-2-HS-glycoprotein (aHSG/fetuin-A) and fibroblast growth factor 21 (FGF21) serum levels in blood of Zucker (Crl:ZUC(ORL)-Lepr^fa^) rats before and 12 weeks after control (SHAM) and ileal transposition (IT) surgery.

Hepatokine concentration (ng/ml)	Pre	Post	Δ^a^	95% CI Δ^b^	p
**SHAM**					
RBP4	3.4 ± 0.6	3.3 ± 0.6	0.0 ± 1.0	(− 1.0; 1.0)	0.945
aHSG	23.9 ± 3.3	25.7 ± 3.4	− 1.9 ± 3.6	(− 5.7; 1.9)	0.259
FGF21	2.4 ± 0.7	2.0 ± 0.5	0.4 ± 0.5	(− 0.2; 1.0)	0.126
**IT**					
RBP4	3.6 ± 0.5	2.5 ± 0.3	1.1 ± 0.7	(0.3; 1.9)	**< 0.05**
aHSG	21.6 ± 2.3	19.9 ± 1.3	1.7 ± 3.5	(− 1.9; 5.4)	0.274
FGF21	1.9 ± 0.5	1.3 ± 0.3	0.7 ± 0.8	(− 0.2; 1.5)	0.093

The CRP results were significantly different before surgery (Table 1: pre IT vs. pre SHAM—24.4 ± 2.3 vs. 16.0 ± 2.8, p < 0.001) thus the values for this parameter are not compared here.

The results are presented as mean ± standard deviation.

Significance for p-value was set at < 0.05.

^a^Δ—difference.

^b^CI Δ—95% coincidence interval.

### Oral glucose tolerance test

**Figure 2:**
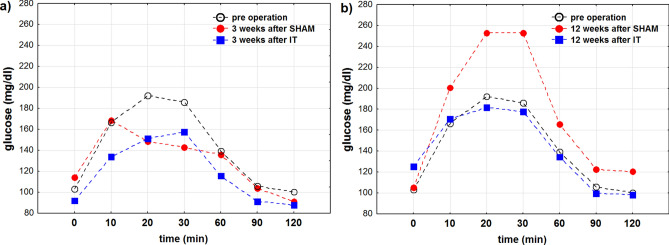
Time profile (min) of mean values of glucose concentration (mg/dl) in 3 weeks (**A**) and 12 weeks (**B**) after operation. For comparison the concentration before operation were marked in black. For reader’s convenience, the points are connected with dashed lines.

The glucose levels measured before the operations and 3, 6, 9 and 12 weeks after IT and SHAM (control) surgery are presented in Table 3. Descriptive statistics and results of two-way analysis of variance with all glucose curves together, regardless of time after IT and SHAM surgery showed that glucose concentration significantly depends on time after glucose administration (Table 3, IT: p < 0.001; SHAM: p < 0.001). Intra-IT and intra-SHAM group analysis showed no significant differences in the profiles of glucose curves during OGTT analysed in consecutive weeks after the procedures (Fig. 2, Table 3, IT: p = 0.349, SHAM p = 0.429).

**Table 3:** Glucose concentration (mg/dl) measured in blood of Zucker (Crl:ZUC(ORL)-Lepr^fa^) rats at consecutive time points during oral glucose tolerance test (OGTT) performed before and 3, 6, 9, 12 weeks after ileal transposition (IT) or control (SHAM) surgery.

Operation type	Blood glucose concentration (mg/dl) after oral administration							AUC (mg/dl)^2^	p_*AUC*_
	0′	10′	20′	30′	60′	90′	120′		
Pre-operation	103.2 ± 14.7	166.2 ± 58.3	192.6 ± 58.4	186.2 ± 68.8	139.2 ± 52.8	105.7 ± 27.4	100.4 ± 18.1	16,682	
**SHAM**									
3 weeks after	114.2 ± 2	168.7 ± 47.5	148.7 ± 41.7	142.5 ± 31.2	135.8 ± 30.8	103.7 ± 6.9	91.3 ± 11.3	15,149	< 0.001
6 weeks after	137.2 ± 40.7	211.2 ± 51.7	220.7 ± 42.9	204.5 ± 25.5	159.7 ± 62.0	123.5 ± 23.0	123.2 ± 22.6	19,437	
9 weeks after	110.7 ± 19.1	174.8 ± 52.4	206.3 ± 49.6	225.2 ± 64.8	144.7 ± 24.6	128.8 ± 11.7	132.0 ± 12.4	19,053	
12 weeks after	105.3 ± 14.0	200.5 ± 26.0	253.2 ± 48.0	253.2 ± 46.5	165.7 ± 31.7	122.5 ± 6.7	120.5 ± 14.0	20,579	
**IT**									
3 weeks after	91.7 ± 16.3	133.4 ± 36.0	151.3 ± 27.7	157.7 ± 29.0	115.9 ± 39.7	91.3 ± 16.9	88.0 ± 8.6	13,406	< 0.001
6 weeks after	111.0 ± 16.9	161.0 ± 20.4	166.8 ± 21.0	150.2 ± 30.9	123.3 ± 22.0	100.0 ± 13.9	95.5 ± 8.9	14,969	
9 weeks after	89.3 ± 12.9	133.7 ± 25.4	158.8 ± 24.8	170.3 ± 28.0	102.2 ± 53.6	92.2 ± 17.6	93.8 ± 6.1	14,016	
12 weeks after	125.0 ± 20.5	170.5 ± 50.5	181.5 ± 59.3	177.7 ± 62.1	134.2 ± 56.0	99.8 ± 29.0	98.0 ± 29.1	16,188	

The results are presented as mean ± standard deviation.

*AUC* area under curve.

For better understanding, the effects of the surgery at two points in time: 3 weeks and 12 weeks after the procedures were selected and the OGTT results for these point times were compared.

#### 3 weeks after surgery

Descriptive statistics and results of two-way analysis of variance for inter-group comparison between rats at 3 weeks after IT and SHAM surgery showed significant differences in glucose concentration depending on time after the glucose administration (p < 0.001) and differences in the profiles of all studied groups in relation to time after the surgery (p < 0.05). Additionally, a difference in the area under the curve (AUC) was observed: the AUC before the operation was 16,682 (mg/dl)^2^ whereas the AUC after IT and after SHAM surgery was lower—13,994 (mg/dl)^2^ and 15,149 (mg/dl)^2^, respectively (Table 3).

#### 12 weeks after surgery

Statistically significant differences in glucose concentrations in relation to time after glucose administration (p < 0.01) were found at that point. There were no significant changes in the profiles of the glucose curves at 3, 6, 9 and 12 weeks after the surgery (p = 0.111). All glucose curves presented a similar shape and trends in glucose concentrations—increase and decrease—at similar time points. The AUC for rats after IT surgery was 16,188 (mg/dl)^2^ and 16,682 (mg/dl)^2^ for rats before the procedures, whereas for rats after the SHAM procedure it was higher—20,579 (mg/dl)^2^ which proves the beneficial effects of the IT procedure (Table 3).

Multiple comparisons in contrast analysis of glucose concentration measured at consecutive time points during oral glucose tolerance test (OGTT) in blood of Zucker (Crl:ZUC(ORL)-Lepr^fa^) rats 3 weeks post surgery period show no statistical significane differences between IT and SHAM operation. Whereas, the same comparisons of glucose concentration 12 weeks after surgery showed statistically significant differences in glucose concentration after IT and SHAM procedures only for results recorded at 20 (p_IT vs. SHAM_ < 0.05) and 30 min (p_IT vs. SHAM_ < 0.05) of oral glucose tolerance test (OGTT).

### Fasting glucose, insulin and HOMA-IR analysis

Fasting glucose concentration in the serum depended on time (p < 0.001) but not on the type of surgery applied in the study (p = 0.481) (Table 4). Twelve weeks after the surgery, fasting glucose concentration was significantly lower in rats after the IT procedure when compared to rats after the SHAM procedure (p < 0.05). Insulin concentration in the serum depended on time (p < 0.05) and on the type of surgery (p < 0.05). Its concentration was significantly lower three (p < 0.05) and lowered 12 weeks after the IT procedure in comparison to insulin concentration measured before the procedure. HOMA-IR depended on the type of surgery (p < 0.01). Three weeks after the surgery, HOMA-IR values were significantly higher in rats after SHAM than in rats after the IT procedure (p < 0.001) (Table 4).

**Table 4:** Fasting glucose (mmol/l) and insulin (µU/ml) concentrations mesured in blood, and HOMA-IR of Zucker (Crl:ZUC(ORL)-Lepr^fa^) rats before, 3 weeks and 12 weeks after control (SHAM) or ileal transposition (IT) surgery.

Parameter	Surgery type	Pre-operation	3 weeks after	12 weeks after	p_operation_	p_time_	p_interaction_	p_before vs. 3 weeks_	p_before vs. 12 weeks_
Glucose fasting (mmol/l)	SHAM	5.3 ± 0.9	4.7 ± 0.7	4.3 ± 0.3	0.481	< 0.001	0.385	0.215	0.066
	IT	5.6 ± 1.5	4.1 ± 0.5	3.9 ± 0.3				< 0.05	< 0.01
	p_SHAM vs. IT_	0.723	0.154	< 0.05				–	–
Insulin (µU/ml)	SHAM	103.7 ± 20.6	149.1 ± 37.1	145.1 ± 33.7	< 0.05	< 0.05	0.252	< 0.05	0.074
	IT	89.1 ± 4.8	96.6 ± 5.6	131.3 ± 53.6				0.661	0.093
	p_SHAM vs. IT_	0.159	< 0.05	0.615				–	–
HOMA-IR	SHAM	24.6 ± 6.4	31.5 ± 6.2	27.6 ± 6.0	< 0.01	0.965	< 0.05	0.093	0.413
	IT	23.6 ± 5.7	17.5 ± 1.4	21.7 ± 5.3				0.134	0.618
	p_SHAM vs. IT_	0.769	< 0.001	0.105				–	–

The results are presented as mean ± standard deviation. Significance for p-value was set at < 0.05.

### Total lipids content analysis

Total lipids concentration in muscles and liver depended on the type of surgery performed (p < 0.001) (Table 5). Total lipid concentration in muscles and liver of SHAM-operated rats was the same before and after the surgery (p = 0.535 for muscles; p = 0.784 for liver), while for IT-operated rats total lipids concentration was significantly lower after the IT procedure (p < 0.001 for both tissues). Both SHAM and IT procedures did not affect the total lipids concentration in the subcutaneous and visceral fat tissue (p = 0.950, p = 0.370, respectively, Table 5).

**Table 5:** Total lipids concentration in muscles, liver, subcutaneous and visceral fat of Zucker (Crl:ZUC(ORL)-Lepr^fa^) rats before and 12 weeks after control (SHAM) or ileal transposition (IT) surgery.

Total lipid concentration in tissues (μg/mg)	Pre-operation	After SHAM	After IT	p_ANOVA_	p_before vs. SHAM_	p_before vs. IT_
Muscles	47.0 ± 7.4	44.4 ± 2.5	27.8 ± 2.0	< 0.001	0.535	< 0.001
Liver	69.8 ± 6.0	72.0 ± 9.3	45.8 ± 3.5	< 0.001	0.784	< 0.001
Subcutaneous fat	697.5 ± 41.1	690.5 ± 40.0	694.2 ± 31.5	0.950	–	–
Visceral fat	698.2 ± 58.9	720.9 ± 67.9	656.1 ± 101.2	0.370	–	–

The results are presented as mean ± standard deviation. Significance for p-value was set at < 0.05.

### Liver histology

**Figure 3:**
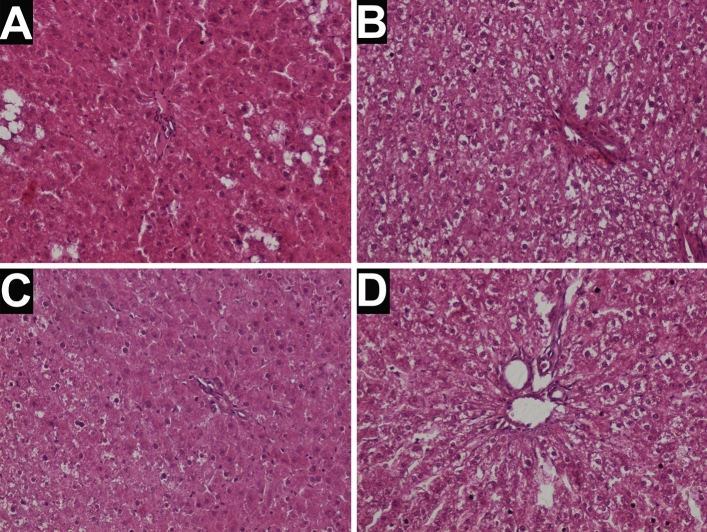
Histological image of liver of Zucker (Crl:ZUC(ORL)-Lepr^fa^) rats after 12 weeks: (**A**) and (**B**) after control (SHAM) surgery; (**C**) and (**D**) after ileal transposition (IT) surgery. H&E staining, ×200.

**Figure 4:**
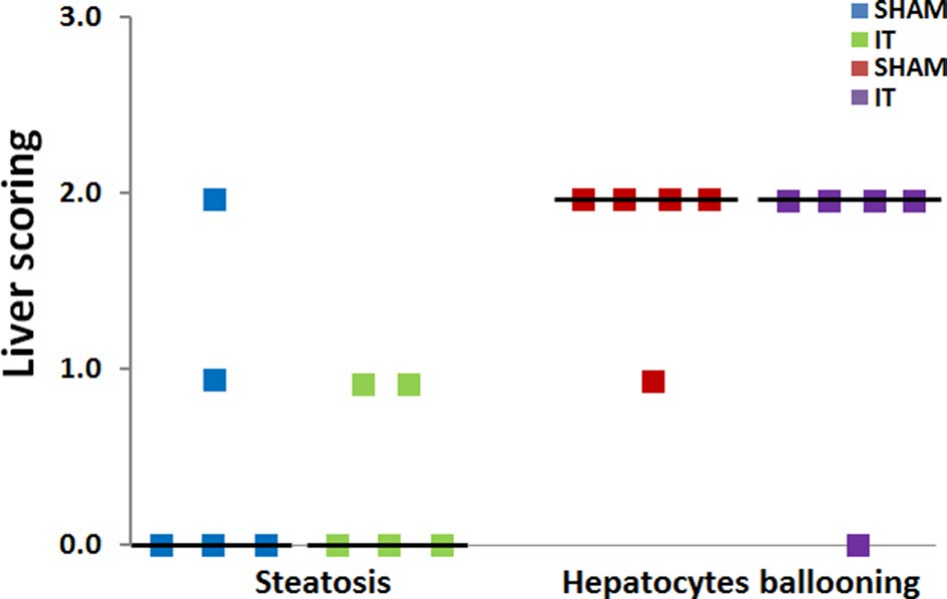
Average steatosis and hepatocytes ballooning in liver samples of Zucker (Crl:ZUC(ORL)-Lepr^fa^) rats 12 weeks after control (SHAM) surgery or ileal transposition (IT). Results are presented as average scoring ± SEM (standard error of measurement) (n = 5).

Histopathological analysis showed a large number of ballooned hepatocytes within hepatic lobules in the livers of rats after the SHAM procedure and only one liver sample with mild steatosis features. Livers of rats after the IT procedure showed evident hepatocyte ballooning (with only one sample with the reduced ballooning process). Only one liver sample from in the IT operated rats showed mild steatosis features (Table 6, Fig. 3). We did not observe any noticeable lobular inflammation or fibrosis in liver samples from both groups of rats. Taking into account all analyzed samples, there were no significant differences between the tested groups (Figs. 3, 4) however the low number of animals in both studied groups (n = 5) may hinder this result.

**Table 6:** Histopathological assessment of Zucker (Crl:ZUC(ORL)-Lepr^fa^) rats liver samples collected from animals 12 weeks after ileal transposition (IT) or control (SHAM) surgery.

Group	Rat no	Steatosis	Hepatocyte ballooning
SHAM	1	1	2
	2	0	2
	3	0	1
	4	2	2
	5	0	2
IT	1	1	0
	2	0	2
	3	1	2
	4	0	2
	5	0	2

## Discussion

Here we present the effects of the ileal transposition (IT) on retinol-binding protein-4 (RBP4), α-2-HS-glycoprotein (aHSG/fetuin-A), fibroblast growth factor 21 (FGF21), and C-reactive protein (CRP) levels in plasma of obese Zucker (Crl:ZUC(ORL)-Lepr^fa^) rats. We report a significant reduction in the plasma levels of selected hepatokines 3 months after IT surgery when compared to the control group of rats that underwent control (SHAM) procedure. The beneficial effect of IT on hepatokines levels was also observed in the intra-group analysis but this change was not statistically significant for FGF21, aHSG and CRP plasma levels. The individual responses of the animals and quite high standard deviation of measured parameters can probably explain this insignificance of the observed changes. On the other hand, HOMA-IR assessed in serum and total lipids concentration measured in the soleus muscle, liver depended on the type of surgery and, in case of total lipids concentration in the mentioned tissues, on time after the surgery.

To the best of our knowledge, there is no data on the effects of IT surgery on RBP4 levels in plasma. We report the significantly lower RBP4 post operative plasma levels in rats after IT procedure when compared to its plasma levels in rats before IT surgery and to the RBP4 plasma level of rats after control (SHAM) surgery. Decreasing RBP4 plasma level contributed to insulin sensitivity improvement in patients after bariatric surgery^28^ and reversely, its substantially increased level signalled the systemic insulin resistance development in humans^29^. In mice, increased levels of plasma RBP4 led to impaired glucose uptake into skeletal muscle and increased glucose production by the liver, whereas lowered plasma RBP4 levels greatly enhanced insulin sensitivity in these tissues^29^. Our results are in line with the research reporting a decrease in RBP4 plasma concentration 12 months following bariatric surgery in 63 obese women^30^. Other research reported that RBP4 systemic levels in obese subjects were significantly decreased after substantial body weight reduction associated with different types of bariatric surgery^15,31,32^ proper dietary plan, physical activity or pharmaceutical interventions^33,34^. During the IT procedure, the isolated segment of ileum is transposed to the jejunum and the intestinal tract retains physiological length but the distribution of endocrine cells along the gut is altered^24,35^. In our research, we observed a reduction in the body weight of animals after IT in comparison to animals after SHAM surgery which may explain the reduction in RBP4 plasma levels and confirm the beneficial effects of the IT protocol. Other scientists reported the opposite situation that bariatric surgery did not affect RBP4 plasma levels. Swarbrick et al*.* reported that 12 months after *Roux-en-Y* gastric bypass (RYGB) performed on 19 severely obese women, the RBP4 plasma levels were the same when compared to pre-surgical levels, despite marked weight loss and improved insulin resistance^36^. Other researchers postulated that a substantial reduction in the mass of adipose tissue and plasma lipid parameters influenced the RBP4 plasma levels rather than insulin sensitivity or inflammatory markers^30^. A decrease in RBP4 levels was only observed after surgically induced weight loss accompanied by relevant reductions in body fat^30^. Since our experiment was performed on Zucker rats with obesity induced by a mutation in the leptin receptor, we suggest that the body mass reduction obtained by IT procedure was substantial enough to lower RBP4 levels in animals with endogenous obesity.

Similarly to the above-mentioned RBP4, no known studies analyse the effect of IT protocol on the aHSG/fetuin-A concentration in plasma, however, the literature repeatedly describes the impact of other bariatric protocols on this hepatokine concentration. Jüllig et al*.* observed a much greater decrease in aHSG/fetuin-A concentration after gastric bypass than after sleeve gastrectomy. Authors suggested that excluding the part of the duodenum from the intestinal passage during the gastric bypass affects the activity of the intestinal microbiota that is significantly involved in the regulation of aHSG/fetuin-A concentration. Sleeve gastrectomy only significantly reduced aHSG/fetuin-A levels^37^. Brix et al*.* showed a beneficial effect of *Roux-en-Y* gastric bypass surgery, again surgery with the partial exclusion of the intestinal passage, on the aHSG concentration^8^. Decreasing aHSG concentration after weight loss is considered a marker of good bariatric effect after surgeries involving gastric bypass^8,37^. aHSG concentration also positively correlated with changes in waist-to-hip ratio (WHR) and body mass index (BMI) 12 months after gastric bypass^10^. In our study, we observed a beneficial effect of IT protocol on the aHSG/fetuin-A concentration measured in plasma 12 weeks after surgery when compared to aHSG concentration in plasma of rats that underwent SHAM (control) procedure. Although the obtained results are statistically insignificant, we have observed some further trends: lower, but not significant, aHSG concentration was measured after IT surgery when compared to pre IT values. We hypothesise that it would be possible to reach a statistically significant change by increasing the number of animals included in the study. In our previous study, we showed that the aHSG/fetuin-A plasma levels and the Ahsg mRNA expression in the liver were lower in rats after bariatric surgery, even though the study included animals with diet-induced obesity and studied effects of different bariatric procedure: duodenal-jejunal omega switch (DJOS)^15^. In the currently presented study, we observed lower sHSG/fetuin-A concentration in rats after IT procedure when compared to concentration level before the IT surgery. On the contrary, comparing the aHSG plasma levels before and after SHAM (control) surgery we observed the increase in the concentration of this hepatokine after the SHAM procedure. SHAM is a surgical intervention without transposition of the ileum and does not have a therapeutic purpose. Since the body weight of the animals that underwent SHAM procedure was increasing during the experiment, the increase in aHSG concentration in the plasma of these animals is understandable.

FGF21 was not analysed in the scientific literature in the context of IT. Liu et al*.* reported the effects of duodenal-jejunal bypass and sleeve gastrectomy and observed a significant decrease in FGF21 concentration in rats after both types of surgeries^16^. Pop et al*.* reported a similar tendency in human subjects after *Roux-en-Y* gastric bypass surgery^38^. Although our study did not show statistically significant differences in FGF21 concentrations, as in the case of aHSG (fetuin-A), noticeably lower concentration of this hepatokine is associated with the state after IT surgery, when compared to its concentration in rats before IT and in these that underwent SHAM procedure. Based on these results, we may conclude that the IT procedure leads to the beneficial effects: reducing levels of hepatokines circulating in plasma. The lack of statistically significant differences between pre- and post IT hepatokines levels can result from individual differences between animals included in the study. We based the number of rats subjected to the experiment on Russell's 3R theory stated in 1959: we used the smallest possible group of animals that enabled us to obtain comparable results. But with such a small number of animals, individual changes can have an impact on the test result^39^. Thus the number of animals included in the study may be also considered the main limitation of our research.

Although Zucker rats partially simulate human metabolic syndrome (obesity, insulin resistance, dyslipidemia, hyperinsulinemia, and liver), this animal model still has some disadvantages. Because leptin receptor mutations are rare in humans, Zucker rats may not reflect the clinical and pathological circumstances observed in humans. Furthermore, Zucker rats do not naturally develop steatohepatitis, are resistant to liver fibrosis, and require additional interventions to induce the progression of steatosis to NASH. FGF21 is involved in a complex interplay with the adipokines, leptin, and adiponectin. In mice lacking either of these adipokines, the beneficial metabolic effects of FGF21 are diminished^40,41^. Thus, the impact of FGF21 on the reduction of liver fat concentration in Zucker rats after IT may be also changed.

The decrease in hepatokines plasma levels seems to be a very good prognostic factor of IT surgery. Nevertheless, it should be remembered that Zucker rats used in the experiment were not healthy: leptin receptor mutation evokes many disorders in itself, not only obesity. The response to surgery in organisms with endogenous obesity is hindered when compared to those with diet-induced obesity. For such animals, namely for animals with endogenous obesity, improving the results of hepatokines and thus improving insulin and glucose metabolism may be considered an optimal achievement.

This study also included the analysis of CRP concentration, considered here as a general inflammation indicator. CRP level in rats after IT surgery was significantly lower than in rats after the SHAM procedure. Obesity itself has been recognized as a factor leading to chronic inflammation, which can directly affect CRP^42^.

When comparing CPR levels before and after the procedures, we noticed that in the group of rats selected for IT surgery, the CRP level was higher before the surgery whereas in the group of rats selected for the SHAM procedure the CRP level was higher after the procedure. Noticeably, the CRP levels in rats selected for both procedures were different even before the procedures, with higher value measures for rats selected for IT surgery. The rats subjected to the study were randomly assigned to the IT and SHAM groups. All animals were kept in the same conditions, having unlimited access to food and water. Animals were not having any signs of acute inflammation or disease. There were no unusual or significant events that occurred during acclimatization time. The 7-week old Zucker (Crl:ZUC(ORL)-Lepr^fa^) rats, upon arrival in the laboratory, were not obese but still, the metabolic profiles of these animals were changed due to the mutation in the leptin receptor. Since both groups were kept in the same conditions and were not subjected to any intervention, it can be assumed that this difference resulted from individual variation. That is why we postulated that the significant differences in the CRP levels before surgery were related to the individual differences resulting from their impaired metabolic processes. We consider this fact as one of the limitations of our study that should be supported by additional analyses.

IT procedure had a significant impact on glucose tolerance in operated rats. Our results show, that time after glucose administration and time after the surgery are significant factors influencing glucose metabolism. Rats after IT procedure showed reduced AUC_OGTT_ at 3 and 12 weeks post IT surgery when compared to rats that underwent SHAM procedure at the same time points. In the same group of rats, we observed that fasting glucose concentration depended on the time after the surgery and it was significantly reduced at 3 and 12 weeks after the IT procedure. HOMA-IR and insulin levels value was significantly lower 3 weeks and declined after 12 weeks after the IT surgery. The positive impact of IT surgery and the ameliorated glucose metabolism was previously reported in Zucker^43^ and Goto-Kakizaki (GK)^44^ rats. We argue that IT surgery leading to subtle body weight reduction is also improving tissue-specific glucose metabolism.

We observed significant differences in the body weight between the IT and SHAM operated rats in the 4th, 6th, 8th and 10th week after the procedures. Ikezawa et al. demonstrated that IT surgery notably reduced the eWAT mass of OLETF (Otsuka Long-Evans Tokushima Fatty) diabetic rats^45^. A study by Yan et al*.* on GK rats also showed reduced body weight, fat mass, and food intake, improved glucose metabolism, and increased insulin sensitivity after IT procedure^46^. On the other hand, other IT studies carried out on Zucker rats^43,47^ and UCD-T2DM rats^48^ revealed no impact of IT procedure on body weight. In our experiment, we observed reduced total lipid content in the soleus muscle and liver tissues but not in the visceral and subcutaneous fat tissue of rats that underwent IT surgery when compared to the pre-operated animals. The reduced fat tissue and increased glucose tolerance after IT procedure were previously reported in several rodent based animal models such as GK rats^49^, OLETF diabetic rats^45^, UCD-T2DM rats^48^, and Sprague Dawley high-fat diet (HFD)-induced obese (SD-DIO) rats^50^. In our study at each time point, the body weight of rats after IT was lower than of those after SHAM. Due to the nature of the Zucker (Crl:ZUC(ORL)-Lepr^fa^) rats and the fact that IT surgery does not involve a reduction of the stomach and/or exclusion of any part of the intestines from the food passage, we did not expect any significant reduction in the body weight after the surgery. A significant difference in the body weight of IT operated rats for these time intervals may be due to individual variability and longer recovery time after surgery. The SHAM procedure, during which the incisions are made only in analogous places to minimise interference in the intestines functioning, can lead to faster regeneration and thus to faster return to normal digestion. Both in the first month after surgery and beyond the 10^th^ week, the differences in the body weight of rats are no longer statistically significant. In the initial period, it may be a similar reaction of organisms to the procedure per se, while in the later period adaptation to new anatomical conditions of the gastrointestinal tract. The selection of rats with a leptin receptor mutation is significant, since Zucker (Crl:ZUC(ORL)-Lepr^fa^) strain is considered to represent metabolic syndrome, the weight loss itself seems to be a much bigger challenge than in primarily non-obese organisms.

Our histological analysis showed the same level of liver steatosis and ballooning in animals that underwent IT or SHAM surgery. Obesity and insulin resistance promote hepatic steatosis by increasing free fatty acids (FFA) delivery to the liver. We observed no steatosis or mild steatosis in the liver of analysed rats. First of all, that might be connected to the low number of animals included in the histological analysis (n = 5). Second of all, the biopsy of the liver was performed in the distal part of the left, lateral lobe (using transverse and longitudinal-vertical section). The obesity of the Zucker (Crl:ZUC(ORL)-Lepr^fa^) rats results from the leptin receptor mutation, which in consequence causes polyethiological obesity. Some authors report that the *Lep*^*ob*^/*Lep*^*ob*^ (ob/ob) mice show mild to severe steatosis at 12 weeks of age, but ballooning and lobular inflammation remain absent even after 20 weeks of age^51,52^. The other studies in the Zucker rat model report that macro/microvesicular steatosis is diffused and located mainly in the periportal area^53^.

In our experiment, we observed mild steatosis, moderate ballooning, and lack of inflammation. It is generally accepted that the genetic mutation alone is not sufficient for worseing of hepatic conditions and a second stimulus is needed. The second stimulus usually takes the form of a chemical challenge (including lipopolysaccharide, CCL_4_, and TAA administration) or dietary measures (including methionine- and choline-deficient MCD diet and HFD)^54,55^. Given that, the second factor stimulating degenerative processes in the liver, such as steatosis and inflammation, might be oxidative stress. In our other study, we observed an increase of TAC and TOS in IT-operated animals comparing to SHAM-operated and control ones. This result might support the evidence that the observed, after IT procedure, reduced oxidative stress may substantially ameliorate insulin resistance and neutralize the negative consequences of obesity.

We found very little differences between samples from rats that underwent SHAM (control surgery) and rats that underwent the IT procedure. This may result from the characteristics of the IT procedure: transplantation of a selected fragment of the ileum to the proximal part of the jejunum does not affect the intestinal absorption surface morphology but can significantly affect the absorption processes both in the interponate and in the remaining parts of the intestine^56,57^. In our previous study, we reported significant changes in the morphology of intestines after IT procedure: increased villus length, decreased crypt depth, changes in the thickness of the mucosamuscularis-serosa (MMS)^24^. The previous paper also reported that rats after IT procedure, when compared to SHAM operated rats, expressed cellular hyperplasia with the increased mitochondrial density in the transposed ileum segment and microvillus degeneration in jejunum regions. The main limitation of our previous study was a lack of biochemical and molecular analysis, which would give information on whether those morphological changes are beneficial or deleterious for IT animals. Knowing that the amount of nutrients consumed by rats is rather the same (both before and after the procedure in both groups), we assume that the amount of absorbed substances does not differ between IT and SHAM operated rats. Studies on diet-induced obese rats that underwent duodenal-jejunal omega switch (DJOS) showed an improvement in the steatosis grade in rats maintained on a high fat diet before and after DJOS surgery^58^. However, DJOS treatment limited the surface for nutrients absorption as this procedure excludes the duodenum from the intestinal passage. It seems that ileal transposition, which does not affect the length of the intestinal passage, is therefore not relevant for fatty liver prevention and treatment. Perhaps longer observation would allow drawing more precise conclusions.

## Conclusions

Ileal transposition (IT) reduces hepatokines’ plasma concentrations but does not improve the liver glucostatic function of the Zucker (Crl:ZUC(ORL)-Lepr^fa^) rats.

## Materials and methods

### Animals, diet, and study design

The study was conducted according to the Guide for the Care and Use of Laboratory Animals (Directive 2010/63/EU) and with the Local Ethics Committee approval of all experimental procedures (58/2014). Local Ethics Committee at the Medical University of Silesia in Katowice approved all experimental protocols. Fourteen male, obese, 12-week-old Zucker rats (Crl:ZUC(ORL)-Lepr^fa^) (Charles River Breeding Laboratories, Wilmington, Mass, USA) were used in the experiment. The animals were kept under controlled conditions: 12:12 light/dark cycle, 70 ± 1% humidity, unlimited access to water and standard rat food (24% protein, 4.9% fat, 7% crude ashes, 4.7% crude fibre, lysine13.6 g/kg, methionine 4.5 g/kg, calcium 12 g/kg, phosphorus 8.3 g/kg; Provimi Kliba AG, Switzerland).

After one week of acclimatisation, the animals were randomly assigned to two experimental groups: SHAM—control surgery group (n = 7) and IT—ileal transposition surgery group (n = 7) and underwent respective surgery.

### Ileal transposition and SHAM surgery

**Figure 5:**
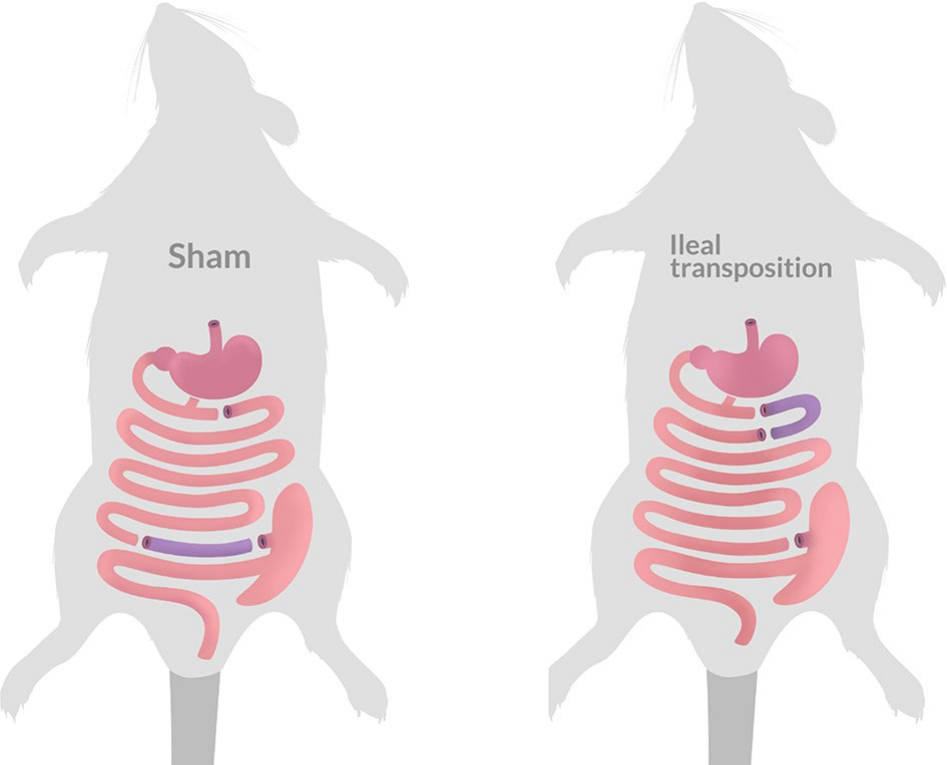
Scheme of ileal transposition (IT) and control (SHAM) surgery performed in Zucker (Crl:ZUC(ORL)-Lepr^fa^) rats. Detailed description of each type of surgery can be found in the “Materials and method” section.

The surgery procedures were performed as previously described by Grüeneberger et al*.* and Stygar et al*.*^43,59^. Briefly, 2% isoflurane with 2 l/min oxygen flow under spontaneous breathing was used to induce and maintain anaesthesia; analgesia was performed with xylazine (5 mg/kg b.w., ip; Xylapan, Vetoquinol Biovet, Poland) and antibiotic prophylaxis with gentamicin (5 mg/kg b.w., im, KRKA, Poland). After an abdominal midline incision (length 4–5 cm) was performed, the Bauhin’s valve was identified. Then 50% of the distal ileum was located and transected. The ileal continuity was restored by an end-to-end extramucosal anastomose using PDS 6/0 (Ethicon Endo-Surgery Inc. USA) and the transposed segment was excluded. Then, the ligament of Treitz was identified and the jejunum was divided 5 cm aborally. The transposed segment of ileum was inserted in an isoperistaltic fashion and two end-to-end anastomoses were performed (Fig. 5). For control (SHAM) surgery, transections were performed at all three analogous points, anastomoses were completed correspondingly, nevertheless without IT (Fig. 5). Fascia and skin closures were performed with a continuous suture using Monocryl 4/0 and Vicryl 4/0. After the surgery, all rats were kept on a liquid diet for 24 h (Nutrison, Nutricia, Poland).

### Blood samples collection, hepatokines, CRP, insulin, and fasting glucose analysis

Under anesthesia and analgesia described previously, blood for hepatokines, CRP, insulin, and fasting glucose analysis was collected from the abdominal aorta into tubes containing 10 μl of EDTA (Sigma-Aldrich, St. Louis, Mo, USA) just before and 12 weeks after the surgery. Then it was centrifuged at 4000 rpm for 10 min at 4 °C and plasma samples were collected and stored at − 80 °C until analysis.

All concentrations were measured in duplicates. Hepatokines, insulin and CRP concentrations were measured using ELISA kits (Cloud-Clone Corp., USA). More specifically: retinol-binding protein-4 (RBP4)—kit no. SEA929Ra with detection range of 0.312–20 ng/ml and minimum detectable dose of < 0.151 ng/ml; α-2-HS-glycoprotein (aHSG/fetuin-A)—kit no. SEA178Ra, with detection range of 6.25–400 ng/ml and minimum detectable dose of < 2.55 ng/ml; fibroblast growth factor 21 (FGF21)—kit no. CEC918Ra with detection range of 12.5–200 pg/ml and minimum detectable dose of < 4.9 pg/ml); insulin—kit no. CEA448Ra with detection range of 123.5–10,000 pg/ml and minimum detectable dose of < 49.3 pg/ml; CRP—kit no. SEA821Ra with detection range of 0.78–50 ng/ml and minimum detectable dose of < 0.34 ng/ml. Fasting glucose concentration was determined using the glucose oxidase method (Sigma-Aldrich Kit, USA).

### HOMA-IR

HOMA-IR was then calculated as fasting glucose (mmol/l) × fasting insulin (μU/ml)/22.5. A homeostatic model assessment of insulin resistance (HOMA-IR) was calculated according to Matthews et al*.*^60^.

### Oral glucose tolerance test

The oral glucose tolerance test (OGTT) was performed before the surgery and every 3 weeks: in the 3rd, 6th, 9th and 12th week after the operation. As the gastric emptying in the initial postoperative period may be hindered and the intestinal passage may be slowed down, the animals fasted for 12 h (with unrestricted access to water) before the OGTT to avoid the effect of potentially residual food on the test result.

For OGTT purposes, the anaesthesia was induced using 2% isoflurane and 2 l/min oxygen flow to facilitate the orogastric tube placement (central venous catheter, Arrow International Inc., Reading, Penn, USA). Analgesia was performed with xylazine (5 mg/kg b.w., ip; Xylapan, Vetoquinol Biovet, Poland). The OGTT was initiated by infusing a 40% glucose solution (Braun Glucose 40%) at 1.5 g/kg dosage via gastric tube. Blood glucose concentration was determined at 0 (fasting), 10, 20, 30, 60, 90, and 120 min of glucose administration, using the glucose oxidase method (Sigma-Aldrich Kit, USA). Blood was collected form the lateral tail vein.

### Body weight measurement

The body weight of rats was measured regularly every 2 weeks, starting from the day of the surgery. It was measured in the most similar conditions possible: at the same time, between 7 and 9 am, before feeding the animals.

### Total lipids contents analysis

Samples (70 mg) of the soleus muscle, liver, subcutaneous and visceral fat were collected from the dissected organs and homogenized with a knife homogenizer in Eppendorf tubes (2 ml). Homogenization was carried out in 1 ml of hexane/isopropanol extraction mixture (3:2 v/v) with the addition of butylated hydroxytoluene.

Total lipids were extracted according to the modified procedure of Kozubek et al*.*^61^. The homogenates were shaken (800 rpm, 20 min, room temperature) and then centrifuged (10,000*g*, 5 min). The obtained supernatant was transferred to a new test tube and treated with 0.5 ml Na_2_SO_4_ and again centrifuged (10,000*g*, 5 min. The upper lipid-containing phase was collected from the solution and evaporated on a thermoblock at 34 °C in a nitrogen stream. The nitrogen atmosphere and the addition of butylated hydroxytoluene were used to protect lipids from the peroxidation process caused by atmospheric oxygen. The obtained dry total lipid extracts were weighed on an analytical balance with an accuracy of 0.1 mg^61^.

### Liver histology

#### Histopathological examination

The liver samples were fixed in 10% buffered formalin, processed and embedded in paraffin. Five μm thick tissue sections were cut. Histopathological preparations were made using haematoxylin–eosin staining (H&E). H&E-stained liver sections were evaluated for ballooning hepatocytes (0—none, 1—few balloon cells, 2—many cells/prominent ballooning) and for hepatic steatosis (0 for < 5%, 1 for 5–33%, 2 for > 33–66%, and 3 for > 66%).

### Statistical analysis

The statistical methodology was previously presented by Stygar et al.^15^. Statistical analysis was performed using STATISTICA 12.5 PL (StatSoft, Cracow, Poland). All tests were two-tailed. Interval data were expressed as mean value ± standard deviation in the case of normal distribution. Distribution of variables was evaluated by the Shapiro–Wilk test and the quantile–quantile plot. The following tests were used to verify hypotheses: parametric test for two independent or dependent samples (the Student’s t-test). Homogeneity of variances was assessed by the Fisher-Snedecore test.

For OGTT data, distribution of variables was evaluated by the Shapiro–Wilk test and quantile–quantile plot, homogeneity of variances was assessed by the Levene's test. The interval data were expressed as a mean value ± standard deviation in the case of normal distribution. For the comparison of data, multivariate repeated-measures ANOVA with contrast analysis at post-hoc were used. Sphericity was tested with the Mauchly's tests. Statistical significance was set at a p-value < 0.05 and all tests were two-tailed. On all glucose plots, the curve for the pre-operation values was marked in black and the same scale was used to facilitate interpretation. The area under the curve was calculated for all OGTT curves.

Data from the histopathological assessment of Zucker (Crl:ZUC(ORL)-Lepr^fa^) rats liver samples are expressed as median with range. Comparisons between medians were done with the nonparametric Mann–Whitney U test.

### Ethical standards

Institutional and national guidelines for the care and use of animals were followed (directive 2010/63/EU). All animal experimental protocols were approved by the Local Ethics Committee (58/2014).

## Data Availability

The original data is available after contact with the corresponding author.
